# Loss of the SWI/SNF ATPase subunits BRM and BRG1 drives lung cancer development

**DOI:** 10.18632/oncoscience.323

**Published:** 2016-11-17

**Authors:** Stefanie B. Marquez-Vilendrer, Sudhir K. Rai, Sarah JB Gramling, Li Lu, David N. Reisman

**Affiliations:** ^1^ Division of Hematology/Oncology, Department of Medicine, University of Florida, Florida, USA; ^2^ Department of Pathology, University of Florida, Florida, USA

**Keywords:** tumor, smarca4, smarca2, lung cancer, swi/snf

## Abstract

Inactivation of *Brg1* and *Brm* accelerated lung tumor development, shortened tumor latency, and caused a loss of differentiation. Tumors with Brg1 and/or Brm loss recapitulated the evolution of human lung cancer as observed by the development of local tumor invasion as well as distal tumor metastasis, thereby making this model useful in lung cancer studies. Brg1 loss contributed to metastasis in part by driving E-cadherin loss and Vimentin up-regulation. By changing more than 6% of the murine genome with the down-regulation of tumor suppressors, DNA repair, differentiation and cell adhesion genes, and the concomitant up-regulation of oncogenes, angiogenesis, metastasis and antiapoptosis genes, caused by the dual loss of Brg1/Brm further accelerated tumor development. Additionally, this Brg1/Brm-driven change in gene expression resulted in a nearly two-fold increase in tumorigenicity in Brg1/Brm knockout mice compared with wild type mice. Most importantly, Brg1/Brm-driven lung cancer development histologically and clinically reflects human lung cancer development thereby making this GEMM model potentially useful.

## INTRODUCTION

The SWI/SNF complex was defined in yeast studies by specific gene mutations that alter two yeast phenotypes, SWItch in mating type (SWI) and Sucrose Non-Fermenting (SNF) [1–4]. A cadre of seven yeast proteins was found to form a single complex, with the inactivation of any one member of the complex yielding similar effects [5]. This observation led to the realization that all subunits of the complex are needed for normal function. In mammalian cells, SWI/SNF is composed of two mutually exclusive catalytic ATPases, Brahma (BRM/SMARCA2) or Brahma Related Gene 1 (BRG1/SMARCA4) and 8-10 BRG1/BRM-Associated Factors (BAFs) [6, 7]. The BAF47 (INI1; SMARCB1; SNF5) subunit was the first to be implicated as a critical tumor suppressor gene whose loss underlies the genesis of malignant rhabdoid tumors [8]. In general, normal SWI/SNF function requires all subunits, which suggests that other subunits might be similarly targeted and silenced in other cancer types. Next Generation sequencing studies have supported this notion by showing that 16-20% of tumors harbor mutated SWI/SNF subunits [9, 10]. Furthermore, immunohistochemistry (IHC) analyses have demonstrated a loss of BRM and BRG1 in 15-35% and 20-50% of human tumors, respectively, with concomitant down-regulation of these subunits in 30% of lung cancer cell lines and 10% of primary lung tumors [9, 11]. In many cases, the mutation rates of these SWI/SNF subunits occur much less frequently than the loss of a given subunit as detected by IHC [10]. As such, mutational inactivation of these subunits in many cases maybe the “tip of the iceberg” in terms of how these different SWI/SNF subunits are targeted, altered and/or silenced during cancer progression. *BRM* and *BRG1* are infrequently mutated (1-2% and 3-6%, respectively) in most human cancers compared with their frequency of loss, which ranges between 15-30 and 20-40%, respectively [9]. Given the essential involvement of SWI/SNF in differentiation, growth control, DNA repair and/or cell adhesion, the loss of one or more subunits would likely impair one or several of these anticancer functions [12, 13].

SWI/SNF is described as a global regulator of gene expression. This complex is recruited to specific DNA regions by a diverse array of proteins including transcription factors and key cellular proteins. At these sites, SWI/SNF functions by shifting the position of histones with the chromatin, which gives transcription factors access to the DNA thereby promoting/repressing gene expression. SWI/SNF functions have been tied to many cellular processes, many of which have been linked to cancer development such as differentiation, development, cell adhesion, growth control, metabolism and DNA repair. Although many different genes have been shown to be linked with SWI/SNF in *in vitro* model systems, the breadth and scope of gene expression impacted by BRG1/BRM loss *in vivo* is unknown. BRG1/BRM proteins have been considered tumor suppressors, as they are known cofactors for both Rb and p53. In BRG1/BRM-deficient cell lines, the induction of p16 or constitutively activated RB fails to inhibit growth. However, RB-mediated growth inhibition can be restored if BRG1 or BRM is induced along with RB. This occurs because BRG1 and/or BRM are known to bind to RB via the LXCXE domain upon which it colocalizes with E2F; this gives E2F access to target genes and the subsequent transcription of E2F-dependent genes. However, the SWI/SNF complex is also known to promote and co-operate with oncogenes such c-MYC and C-JUN to drive growth. Hence, it is not certain whether *BRG1* and/or *BRM* inactivation will promote cancer development or inhibit it.

Targeted murine inactivation of *Baf47* is highly tumorigenic, which supports its role as a tumor suppressor as well as the importance of the disruption of the SWI/SNF complex during the process of tumorigenesis [14]. Brm deficiency in mice causes disruption of cell cycle control as exemplified by the observation that Brm-deficient mice are heavier than wild type animals, and that cells from these animals exhibit abnormal cell cycle control [15]. The inactivation of Brm in the prostate is associated with increased proliferation and the development of castration-resistant epithelial growth [16]. Although a homozygous *Brg1*-knockout is embryonically lethal in mice, a hemizygous *Brg1*-knockout yields breast tumors in 10% of mice after about 1 year [17]. However, given that Brm and Brg1 may functionally substitute for one another [13], individual knockout studies do not completely impair or inactivate the SWI/SNF complex. In particular, the joint loss of Brg1 and Brm, which are required for SWI/SNF function, would likely dramatically affect each of these associated cellular functions and lead to cancer development via a number of mechanisms.

The pursuit of *Brg1* knockout concomitant with *Brm* inactivation is described herein, as an understanding of the impact of BRG1 and BRM loss in cancer is vital to our understanding of cancer development and progression. To this end, the tumors that arose in this system closely and remarkably recapitulate both the histology and pathology that are typically observed in human lung cancer. An understanding of how BRG1/BRM loss drives increased tumorigenesis is underscored by the number genes as well as by the types of gene whose expression inevitable changes due to BRG1/BRM loss. Lastly, the observation of both local and distal metastatic tumors in this system helps to distinguish this model since metastasis is an infrequent event reported in other murine systems.

## RESULTS

### Brg1/Brm loss potentiates the development of malignant tumors

**Figure 1:**
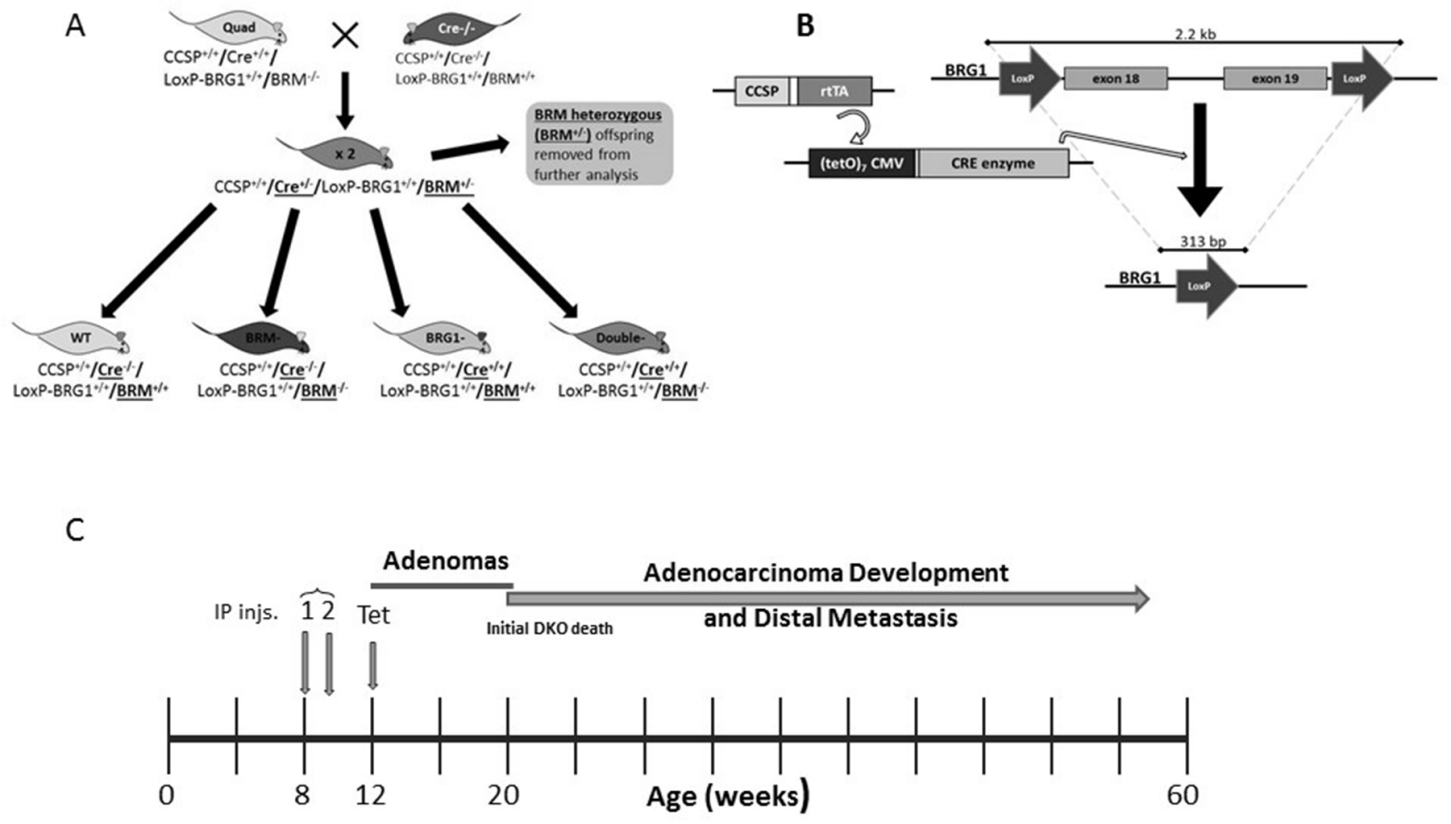
**A.** shows the four different mouse genotypes (wild type, *Brg1*-knockout, *Brm*-null and DKO) and the means by which they were generated. **B.** illustrates the process by which the *Brg1*-knockout mice were generated; *Brg1* exons 16 and 17 were flanked by LoxP sites [45]. Upon tetracycline administration, the Cre enzyme was expressed, which then cleaved the LoxP sites, resulting in a deletion of these two exons. **C.** shows the experimental design of the *Brg1* and *Brm* knockout model, which results in tumor development. Ethyl carbamate and tetracycline administration occurred at 8 and 12 weeks, respectively. Adenoma development occurred at 12 weeks followed by progression and the development of malignant adenocarcinomas beginning at 22 weeks. No further tumors were found in these mice at 60 weeks and thus the experiment was ended.

**Figure 2:**
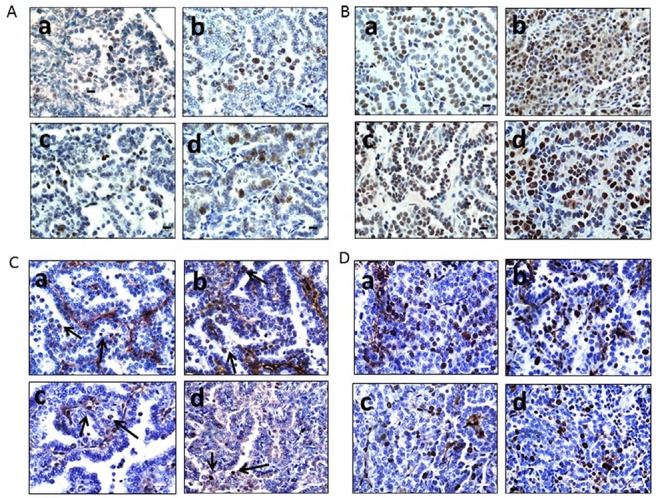
**A.** illustrates Pcna expression by IHC in adenomas from WT (*Brg1*+/+*Brm*+/+) (a), *Brg1* knockout (*Brg1*−/−*Brm*+/+) (b), *Brm*-null (*Brg1*+/+*Brm*−/−) (c) and double KO (*Brg1*−/−*Brm*−/−) (d) mice. **B.** shows the relative Pcna expression in lung adenocarcinomas from DKO (d)>*Brg1*-KO (b), *Brm*-null (c) and > WT (a) mice. Magnification bar (right lower corner) = 40 μm. According to **C.** very few cells express Ki-67 in the nucleus (arrows) in adenomas from a WT mouse (a), *Brg1* KO mouse (b), *Brm*-null mouse (c), and a DKO mouse (d). **D.** shows that, compared with adenomas, a higher percentage of cells express Ki-67 protein (arrows) in adenocarcinomas from a WT mouse (a), a *Brg1* KO mouse (b), a *Brm*-null mouse (c) and a DKO mouse (d). Microscope bar = 20 μM.

To determine the impact of Brg1 and/or Brm loss on cancer development, we generated four molecular phenotypes, as follows: wild type (control: *Brg1*+/+*Brm*+/+), *Brm*-null (*Brg1*+/+*Brm*−/−), *Brg1*- knockout (*Brg1*-KO, *Brg1*−/−*Brm*+/+) and *Brg1*/*Brm*-deficient (double knockout mice, DKO: *Brg1*−/−*Brm*−/−) (Figure 1A). At 8 weeks of age, all mice received an IP injection of ethyl carbamate (urethane) to initiate transformation (Figure 1C). In each of the genotypes, we first observed the development of small lung microadenomas, which were typically <1 mm in diameter when the mice were 12 weeks of age. Previous attempts to inactivate *Brg1* in normal lung tissue resulted in apoptosis, while in contrast, we observed that *Brg1* could be successfully inactivated when lung cells had progressed to adenomas. This is thought to occur because of the frequent Kras mutations induced by ethyl carbamate administration [21, 22] that not only cause the development of adenomas but also the suppression of apoptosis [22, 23]. Hence, we then selectively inactivated (i.e., knocked out) *Brg1* in the lungs of the mice through the induction of Cre recombinase via tetracycline administration (Figure 1B). We confirmed *Brg1* inactivation within the lung (in the adenoma cells that were proliferating during tetracycline administration [24]) of these mice by qPCR (data not shown). When these mice were 16 weeks of age, the microadenomas progressed into readily visible, benign adenomas that measured 2-3 mm in diameter. The differentiation status of these adenomas as well as the number of adenomas did not differ significantly among mice of the four genotypes. The adenomas featured little or no proliferation, as the tumor cells were observed to have minimal immunoreactivity to Pcna (∼12%) (Figure 2A). The progression from adenomas to malignant adenocarcinomas was first observed at 22 and 23 weeks in the DKO and *Brg1*-KO mice, respectively (Figure 1C). The adenocarcinomas could be distinguished from the adenomas by their larger size (>5-10 mm in diameter) and positive staining for Pcna (Figure 2A and 2B) (∼90% positive in adenocarcinomas versus ∼10-12% in adenomas) and Ki-67 (40-45% positive in adenocarcinomas versus 5-8% in adenomas) (Figure 2C and 2D). These features are consistent with those found in previously published reports [25–27].

**Figure 3:**
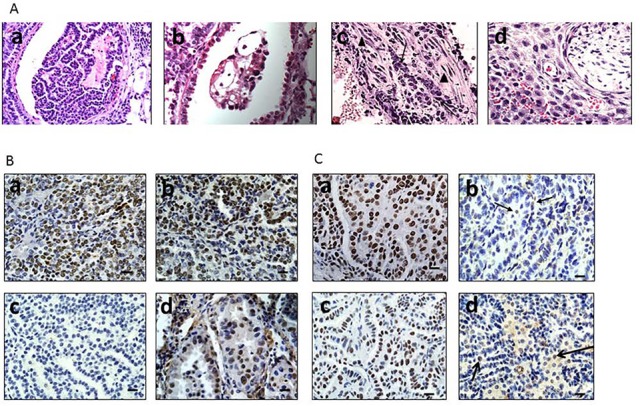
**A.** shows local progression of a malignant adenocarcinoma into a blood vessel (a), a cluster of tumor cells invading a bronchiolar airway (b), the presence of tumor cells in the stroma (large tumor cells, arrow) alongside spindle-shaped fibroblasts (arrowheads) (c) and tumor cells surrounding and invading a nerve (d). **B** and **C.** contain representative images of Brm and Brg1 expression in tumors, respectively, as detected by IHC in tumors from the four genotypes: WT (a), *Brg1*-KO (b), *Brm*-null (c) and DKO (d); arrows indicate Brg1-positive cells within the *Brg1*-KO and DKO tumors.

The adenomas were encapsulated by a distinct boundary, whereas the adenocarcinomas often showed infiltration and extension of tumor cells into the surrounding tissue with vascular invasion, airway spread, stromal invasion and perineural invasion (Figure 3Aa–3Ad). Unlike adenomas, which were observed to be well tolerated even when they numbered as high as ∼30-50 per mouse [20], the development of these malignant lung adenocarcinomas eventually caused visible distress: weight loss >10%, lack of movement, disheveled appearance, and tachypnea. The majority of observed tumors were harvested when one or more of these physical changes were observed as required by IACUC. In addition, a few remaining tumors were observed and harvested at 60 weeks when the number mice that succumbed to these tumors greatly decreased, and at this time, all surviving mice were euthanized to end the experiment. The knockout of *Brm* and/or *Brg1* in each genotype was confirmed by IHC in all tumors (Figure 3B and 3C). While the majority of tumors (>60%) from *Brg1* knockout mice showed near complete loss of Brg1 expression, a subset of tumors ∼20-25% showed a mosaic pattern of Brg1 loss.

### Loss of BRM and BRG1 is associated with loss of differentiation

**Figure 4:**
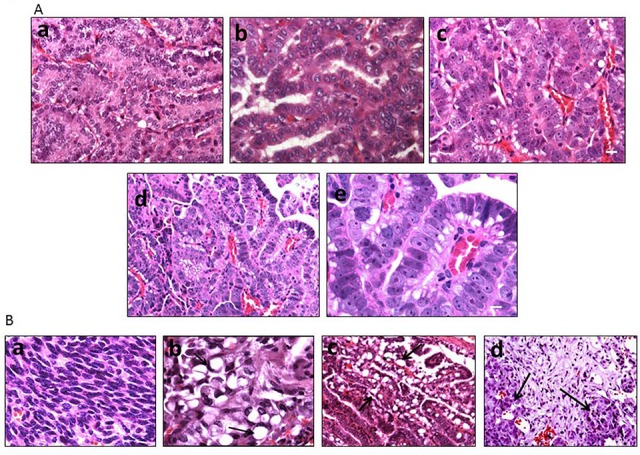
**A.** shows the various differentiation states (well, moderate and poor) as illustrated by H&E staining. The well-differentiated tumor shows well-formed papillary and glandular structures and mild cytological atypia (a). The moderately-differentiated tumor is composed of irregular glandular structures, moderate cytological atypia and occasional mitotic figures (b). The poorly-differentiated tumor shows a predominantly solid growth pattern, marked cytological atypia and increased mitotic figures (c). Low (d) and high (e) magnification of a high-grade tumor with prominent nucleoli and mitotic figures. Magnification bar = 20 μM in a-c; 10 μM in e. **B.** illustrates a tumor with sarcomatoid morphology, the most poorly differentiated variant (a), a cluster of signet ring cells (arrows) (b), mucinous tumor cells with cytoplasmic vacuoles (black arrows) (c), and a pale staining area of fibrosis with tumor cells (black arrows) (d).

As the SWI/SNF complex is known to regulate and induce differentiation in a variety of cells and tissue types [13, 28, 29], we next examined the differentiation state of each of these tumors as a function of Brm and/or Brg1 expression. A histological comparison showed that the loss of expression of BRM, BRG1 or both caused the tumors to become de-differentiated, as shown by tumors that are representative of well, moderate and poor differentiation (Figure 4Aa–4Ac). The inactivation of *Brg1* or *Brm* was observed to elicit a similar de-differentiated state relative to each other, whereas tumors from either *Brg1*-KO or *Brm*-null mice were less differentiated (p<0.05) than WT tumors but were more differentiated than DKO tumors. In comparison, the loss of both Brg1 and Brm resulted in a greater degree of de-differentiation (p<0.05). Specifically, the highest grade of de-differentiation, as exemplified by sarcomatoid morphology (Figure 4Ba), was primarily seen in the DKO tumors, especially in those tumors that invaded and penetrated the chest wall. High-grade morphology, which was characterized by a high nuclear to cytoplasmic ratio, atypical nuclei, prominent nucleoli, frequent mitotic figures, and a solid growth pattern, was most often found in mice with a DKO phenotype (Figure 4Ad–4Ae).

**Figure 5:**
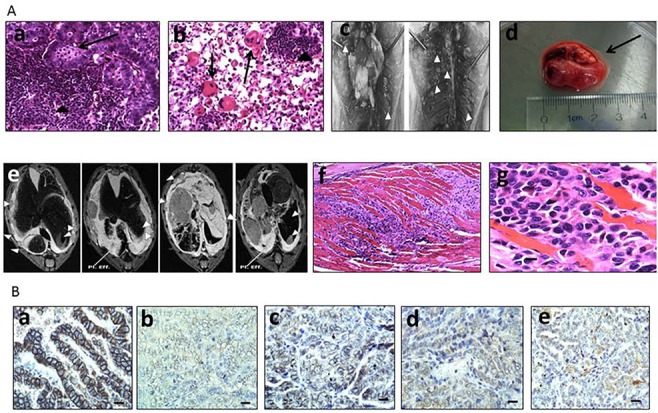
**A.** shows a sheet of lymphocytes (arrowhead) and a cluster of macrophages (arrow) (a), as well as several multinucleated giant cells (arrows) have infiltrated these tumors; arrowhead, lymphocytes (b). Also illustrated is the studding (tip of white triangles) of the ribcage after gross dissection of a DKO mouse with the lungs in place and after the lungs were removed (c). One of the tumors noted in the MRI was removed and examined and was revealed to be ∼2 cm in length and contain a clear fluid indicative of a pleural effusion (black arrow) (d). MRI images of the larger adenocarcinomas of the lungs, the ribcage mets (denoted by white triangle tips), as well as and pleural effusions (white lines) are shown in (e). H&E stain of malignant lung adenocarcinoma cells that have penetrated through the chest cavity into the intercostal muscles of the ribcage in a BRG−/− mouse at low mag 10x (f) and high mag 40x (g). **B.** shows strong E-cadherin expression in a lung adenocarcinoma derived from a WT mouse (a), but much weaker staining in tumors from a *Brg1*-KO mouse (b), a *Brm*-null mouse (c), and a DKO mouse, where E-cadherin expression is almost completely absent (d). There is minimal to no expression of E-Cadherin in a metastatic rib met (e), which was derived from the mouse in (b).

Mouse models that closely resemble or mimic human pathology are highly sought after. Pathologic examination of the tumors that arose as result of *Brg1* and/or *Brm* knockout showed a number of features that are indicative of and that exemplify the complexity of human lung cancer. Multiple tumors from DKO mice also exhibited “signet ring” cells, in which the nucleus is pushed to the periphery of the cells due to the large amount of mucin produced by the tumor cells (Figure 4Bb–4Bc). The development of effective, immune checkpoint inhibitorssuch asPDL-1 (CD274), PD-1 and CTLA-4 has brought to the forefront the importance of the immune system in the treatment of lung cancer. Hence, the observation that a subset of these tumors displayed an extensive infiltration pattern by lymphocytes, macrophages and multinucleated giant cells (Figure 5Aa–5Ab) combined with the known role of the SWI/SNF complex in modulating the immune system (i.e., Brg1 regulation of Pdl1), further argues the potential utility of this model. Other tumors demonstrated fibrosis and extensive desmoplastic reactions, which is in part, indicative of the healthy tissue response to invading tumor cell. Hence, this model recapitulates human lung cancer in a number of different ways thereby potentially making it an important model to be used in future studies.

### BRG1/BRM loss yields local invasion

Another critical feature of human tumors that is not often recapitulated in many if not most murine lung cancer models is the occurrence of documented local and distal spread of tumor cells. To this end, the development of local invasion was observed in a number of these lung tumors. In particular, the majority of tumors that demonstrated local invasion (11/12) were Brg1-deficient (*Brg1*-KO and DKO phenotypes). Specifically, we sacrificed a number of mice that succumbed to early respiratory compromise, as demonstrated by tachypnea and the use of accessory muscles for respiration. Upon opening the chest cavity, these mice were found to have large, bloody pleural effusions (exudative effusions), and further inspection of these mice revealed pleural studding caused by the invasion of these tumors into the pleural cavity (Figure 5Ac–5Ad). To investigate the underlying pathology, magnetic resonance imaging (MRI) was conducted on several severely ill mice that demonstrated tachypnea. In these mice, both lungs were found to be compromised by tumor penetration into the pleural space (Figure 5Ae), which resulted in the formation of pleural studding, and subsequently, the accumulation of pleural effusions (Figure 5Ac and 5Ae). Moreover, some tumors progressed further as these tumors not only penetrated the pleura but also invaded the chest wall; the most poorly differentiated of these tumors demonstrated invasion into the ribs and musculature of the chest wall (Figure 5Af–5Ag), which is also observed in clinical lung cancer [30]. As E-cadherin loss *in vitro* has been linked to Brg1 loss as well as to metastatic behavior, we stained for E-cadherin in the primary tumors as well as in sites within the chest that showed local tumor invasion. Both the primary tumors and the sites of local invasion from Brg1-deficient mice had qualitatively less E-cadherin staining compared with the tumors from Brg1-positive wild type mice (Figure 5Ba–5Be).

### BRG1 and BRM loss promotes cancer development by altering the gene expression profile of tumors

It is known that the SWI/SNF complex is a chromatin modifying complex whose function is to interact with a vast array of key cellular proteins and transcription factors, which opens up/closes certain DNA domains to foster gene regulation. In this capacity, SWI/SNF is a central and required cofactor for successful transcription factor-mediated gene expression. While BRG1 and BRM loss have been studied in detail within cell lines (microarray studies) and in association with certain genes, the overall net effect of BRG1/BRM loss on gene expression in hundreds of molecularly heterogeneous tumor cells is not yet known. To begin to understand scope of genes regulated by Brg1 and Brm and by extension, how Brg1/Brm loss fosters tumor progression, we conducted a microarray experiment that compared the gene expression profile of tumors from DKO and wild type mice. Specifically, we compared the microarray gene expression profile from wild type tumors (n=5) with that of DKO tumors (n=6); each tumor was tested in duplicate. Of the ∼23,000 genes within the murine genome, the loss of Brg1/Brm regulates 6.1% of genes greater than 2-fold and 2.2% of genes greater than 3-fold, which is consistent with gene regulation analyses in other species [31, 32] (Supplementary Table 1). Moreover, 25 genes that are included in the FoundationOne assay to determine genomic profiles of human cancers were found to be regulated by BRG1 and or BRM (Supplementary Table 2). In order to validate these observed microarray results, we conducted qPCR on 101 cancer related genes, and found the positive predictive values for the 2 and 3 fold induced gene to be 87% and 72%% respectively.

As SWI/SNF can have both cancer promoting and inhibitory effects, we sought to determine the specific categories of genes that were up-regulated or down-regulated. To accomplish this, we categorized the functions of over 800 genes that were regulated >2.0-fold by looking at their function as defined in publications listed in NCBI/Pubmed. According to this analysis, specific trends were apparent. Overall 90% of those genes with a defined role in proliferation and/or tumorigenesis were observed to be up-regulated. Similarly, we observed that the vast majority of those genes (>90%) with a defined role in differentiation, tumor suppression, and metabolism were down-regulated. Therefore, the net effect of inactivation of Brg1/Brm was to drive cancer development, rather than block it.

Another important question involves the role of Brg1 and Brm in the specific regulation of cancer development. Do Brg1 and Brm have overlapping and redundant functions where gene dysregulation only occurs when both genes are silenced? Alternatively, do Brg1 and Brm have separate functions that regulate a different spectrum of genes? This issue has been complicated by *in vitro* transfection experiments using strong viral promoters, which yield high and non-physiologic levels of Brg1 or Brm that result in the activation of both Brg1- and Brm-dependent complexes. In order to better understand if cancer-related genes are specifically regulated by Brg1, Brm or both, we next conducted qPCR of 70 genes in tumors from mice of each of the four genotypes. This analysis revealed no single pattern of Brg1/Brm regulation. While some genes such as Adh1, Gadd45a, and Ros1 are more or less equally regulated by Brg1 and Brm, other genes such as Dmkn, Crp, Plau, and Etv-1 are primarily linked to Brm, whereas others such as Ear2, Serpinb2 (PAI-2), Redd1, and Mmp1 are primarily regulated by BRG1 (Tables 1 and 2).

**Table 1:** The loss of Brg1 and/or Brm can foster cancer development via the down-regulation (as measured by qPCR) of 27 potential anticancer genes from at least 8 categories (tumor suppressor, immunity, drug metabolism, DNA repair, differentiation, apoptosis, cell adhesion and metabolism)

Gene Name	Alias	Fold Change (Down)			Gene Function	Other Functions
		*Brm* null	*Brg1* KO	(DKO)		
**Acsm1**	**BUSC1, MASC1**	2.3	3.3	5.9	Metabolism	
**AldoB**	**ALDO2**	3.5	2.7	5.4	Metabolism	
**Sult3a1**	**ST3A1, SULTX2**	3.3	4.8	7.5	Metabolism	
**Cd44**	**MDU2, Pgp1**	5.2	2.3	7.1	Adhesion	
**Cdh1**	**E-Cadherin**	2.2	3.6	5.9	Adhesion	
**Lama4**	**--**	8.0	6.8	8.5	Adhesion	Differentiation
**Lamb2**	**LAMS, NPHT**	4.2	2.6	5.2	Adhesion	
**Lin7a**	**TIP-33, MALS-1**	2.4	4.7	6.5	Adhesion	
**Mmp1**	**CLG, CLGN**	3.2	6.8	8.4	Adhesion	
**Serpina3k**	**RP54, MMCM2**	4.5	4.7	8.1	Apoptosis	
**Tnfsf10**	**Trail, Apo-2l**	4.5	1.8	5.5	Apoptosis	
**Ahsg**	**AHS, A2HS**	3.9	2.4	6.8	Differentiation	
**Dmkn**	**UNQ729, ZD52F10**	7.1	1.4	7.5	Differentiation	Biomarker
**Gdf2**	**BMP9, HHT5**	4.7	1.7	5.7	Differentiation	Suppressor
**Tnk**	**ACK, ACK1**	1.5	2.6	3.9	Differentiation	
**Zfhx4**	**ZFH4, ZHF4**	2.8	3.2	5.7	Differentiation	
**Gadd45A**	**BcDNA, GADD45**	8.5	7.6	8.8	DNA Repair	
**REDD1**	**DDIT4, Dig2**	1.3	6.7	7.5	DNA Repair	Suppressor
**Cyp2e1**	**P450C2E, P450-J**	4.5	3.0	7.0	Drug metabolism	
**Cyp2j**	**--**	1.5	3.1	4.8	Drug metabolism	
**Cyp3a11**	**Pcn, Cyp3a**	1.5	4.7	5.8	Drug metabolism	
**Cyp3a25**	**--**	2.4	4.4	7.2	Drug metabolism	
**Cyp4a10**	**RP1, D4Rp1**	2.4	4.3	6.9	Drug metabolism	
**Ugt2b37**	**0610033E06Rik**	1.4	5.4	5.9	Drug metabolism	
**Ugt3a1**	**--**	1.7	3.9	4.8	Drug metabolism	
**Crp**	**PTX1**	9.3	1.7	10.5	Immunity	
**Ddx58**	**RIG-1 RLR-1**	6.6	2.4	7.2	Immunity	
**Ido2**	**INDOL1**	1.8	4.5	5.7	Immunity	
**Serpina5**	**Pci, PAl-3**	1.3	3.3	4.0	Immunity	
**Adh1**	**--**	5.8	8.1	15.0	Suppressor	
**Atf3**	**LRG-21**	13.2	3.5	14.2	Suppressor	Metastasis
**Btg3**	**ANA, Tob-5**	3.9	3.0	6.6	Suppressor	
**Dcn**	**PGS2, SLRR1B**	3.0	0.7	3.4	Suppressor	
**Dhcr24**	**seladin-1**	1.5	4.0	5.4	Suppressor	
**Dkk3**	**--**	1.1	5.5	6.3	Suppressor	
**Fat2**	**EMI2, FATH2**	1.8	4.0	6.0	Suppressor	
**Prox1**	**--**	1.7	6.8	8.5	Suppressor	
**RARRES3**	**RIG1, TIG1**	1.1	5.7	8.0	Suppressor	
**SP100**	**Lysp100b**	4.5	1.5	6.5	Suppressor	

The gene name and alias (when applicable) are given in columns 1 and 2, respectively. However, the fold induction of gene expression that was observed for *Brm*-null, *Brg1*-KO- or DKO-derived tumors as compared with wild type tumors is given in columns 3, 4 and 5, respectively. The potential gene function and/or other potential gene functions are listed in columns 6 and 7, respectively.

**Table 2:** The loss of Brg1 and/or Brm can enhance cancer development via the up-regulation (as measured by qPCR) of 26 potential cancer-promoting genes from at least 5 categories (angiogenesis, anti-apoptosis, cancer progression, metastasis and proliferation)

Gene Name	Aliases	Fold Change (Up)			Gene Function	Other Functions
		*Brm* null	*Brg1* KO	Doubles (DKO)		
**Itga2**	**CD49B, DX5**	1.4	3.3	4.3	Angiogenesis	Metastasis
**Sema7A**	**Sema-L; semaphorin**	2.3	3.5	4.2	Angiogenesis	Metastasis
**Serpine1**	**PAI-1**	3.4	5.1	8.9	Angiogenesis	Anti-apoptosis
**Ear2**	**Rnase2, Raf3**	1.3	8.7	9.6	Anti-apoptosis	
**Hecw1**	**Nedl1**	2.2	4.4	5.1	Anti-apoptosis	
**Xaf1**	**Fbox39**	4.0	1.7	5.9	Anti-apoptosis	
**Ddx46**	**PRPF5**	9.5	3.5	10.2	Cancer progression	
**Epcam**	**CD326, EGP**, **gp40**	2.1	4.0	5.2	Cancer progression	
**Macc**	**Gm267**	1.7	3.3	5.7	Cancer progression	
**Tmprss6**	**IRIDA**	1.8	7.3	9.7	Cancer progression	
**Dkk1**	**mdkk-1**	1.1	5.4	7.4	Metastasis	
**Itga3**	**CD49C, GAPB3**	4.4	3.1	7.1	Metastasis	
**Pdpn**	**GP38, RANDAM-2**				Metastasis	
**Plau**	**u-PA**	9.3	2.0	8.8	Metastasis	Tamoxifen-Sensitive
**SerpinB2**	**PAI-2**, **ovalbumin**	1.0	6.1	6.3	Metastasis	
**SerpinB5**	**PI-5, Maspin**	1.1	3.2	3.5	Metastasis	
**Serpine1**	**PAI-1**	2.0	3.2	4.8	Metastasis	
**Etv1**	**ER81**, **Etsrp81**	4.9	1.5	6.0	Proliferation	Oncogene
**Hapln1**	**CLP, LP-1**	0.7	1.8	5.3	Proliferation	Oncogene
**MycN**	**Nmyc-1**	4.4	2.3	6.5	Proliferation	Oncogene
**Areg**	**Mcub, Sdgf**	4.1	1.6	4.5	Proliferation	Oncogene
**Grem1**	**Drm**	3.0	1.7	4.6	Proliferation	Oncogene
**Ros1**	**c-ros**	2.6	2.8	5.3	Proliferation	Oncogene
**Mcoln3**	**TRPML3**	2.2	2.3	4.4	Proliferation	
**Maf**	**--**	2.0	6.0	8.1	Proliferation	Oncogene
**Muc1**	**CD227, EMA**	1.4	3.0	3.9	Proliferation	Metastasis

The gene name and alias (when applicable) are given in columns 1 and 2, respectively. However, the fold induction of gene expression that was observed in BRM-null, BRG1-KO or DKO-derived tumors compared with wild type tumors is given in columns 3, 4 and 5, respectively. The potential gene function and/or other potential gene functions are listed in columns 6 and 7, respectively.

The data cancer related-genes shows that Brg1 and/or Brm loss functions to down-regulate genes that are involved in cell adhesion, differentiation, apoptosis, drug metabolism, metabolism, and immunity, whereas Brg1/Brm loss also causes the up-regulation of genes involved in angiogenesis, anti-apoptosis, cancer progression, metastasis and proliferation (Tables 1 and 2). Moreover, the majority (>90%) of genes involved in different types of metabolism such as amino acid, hepatic-steroid, fatty acid/lipid, and xenobiotic/drug metabolism, were down-regulated. Similarly, we observed a down-regulation of over 90% of genes that code for membrane transport proteins (solute carrier proteins). Conversely, a cadre of genes involved in glycolysis was either up-regulated (e.g., Fbp2, Eno2, and G6pdx), while a subset of genes involved in Krebs cycle entry was also noted to be down-regulated (e.g., Pck1, Slc25a13, Pdk1, Pcx, Got1, Got2) (Supplementary Figure 1). While either Brg1 or Brm generally regulates most genes, the loss of both produced a maximal impact in most cases, which implicates a degree of functional redundancy between Brg1 and Brm. Hence, Brg1 and/or Brm loss appears to foster multiple facets of cancer development via an overall change in the cellular gene expression profile that favors the expression of tumor progression genes and the concomitant reduction in the expression of genes that block or thwart cancer development.

### Loss of BRG1 and BRM accelerates the development of tumors and potential metastatic phenotypes

**Figure 6:**
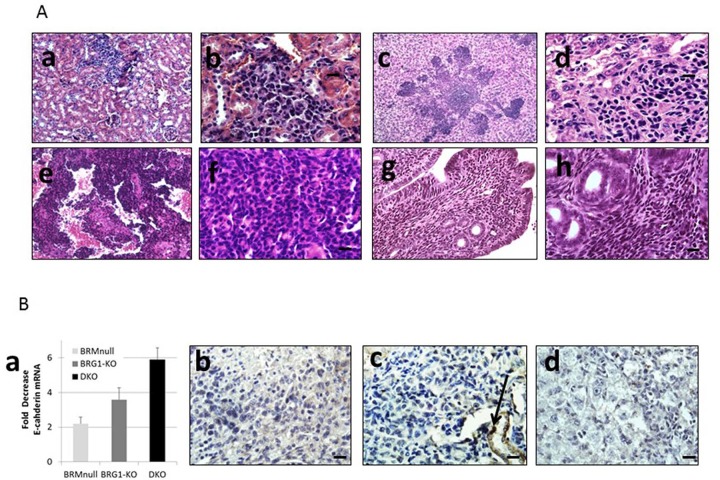
**A.** shows a low (a) and high (b) magnified image of a kidney met from a *Brg1*-KO mouse; (c) and (d) show a liver met from the same mouse; a low (e) and high (f) mag image of a kidney met from a DKO mouse that features a dense sheet of tumor cells; a colon met from a *Brg1*-KO mouse displays spindle-shaped sarcomatoid tumor cells surrounding the normal glandular structures (g-h). **B.** shows the results of qPCR of multiple tumors for E-cadherin and the fold-change of E-cadherin expression in several *Brm*-null, *Brg1*-KO and DKO tumors relative to WT tumors (a). A colon met (b), a kidney met (c) and a liver met (d) from several BRG-deficient mice; the arrow in (c) denotes normal kidney. Magnification bar = 20 μm.

As Brg1/Brm loss changes the gene expression profiles to favor cancer development in conjunction with the effects that Brg1 and Brm loss appears to exert on Rb and p53 function, we would expect that the loss of Brg1 and/or Brm would hasten tumor development. While human NSCLC often involves metastatic disease, most murine tumor models including genetically engineered mouse models (GEMMs) do not involve the development of metastases. In our model, we observed that mice succumbed to complications from increased tumor burden and local tumor metastasis as well as from the development of distant metastases in a subset of these mice. We specifically examined the kidneys, adrenal glands and the liver, as the latter two organs are common sites of metastasis in human lung cancer [30]. We observed adenocarcinoma deposits by gross inspection in about 10% (n=271: all genotypes) of the total population of experimental mice, especially in the liver and kidney and less frequently in the colon (Figure 6A). By microscopic comparison of a subset of 10 cases, the histologic features of the metastatic lesions were similar to those of the corresponding primary lung tumors (Supplementary Figure 2). Moreover, in the analysis of a subset of each genotype, the occurrence of local invasive and distant metastases was primarily observed in the DKO mice (∼16% n=14/87of all DKO mice) and in the *Brg1*-KO mice (∼13% N=12/91 of all BRG1-KO mice), but was not largely observed in mice of the other two genotypes. This suggests that Brg1 loss predisposes a tumor to metastatic behavior. This assertion is supported by changes in gene expression detected in Brg1-negative tumors compared with WT tumors (Tables 1 and 2). Since SWI/SNF regulates E-cadherin expression [33, 34], whose loss can drive de-differentiation as well as metastatic behavior, we stained for E-cadherin in tumors from each genotype (Figure 5Ba–5Be). We observed that the loss of BRG1 in these lung tumors correlated closely with the loss of E-cadherin, which is consistent with findings in BRG1-deficient cell lines [34]. Moreover, by qPCR, we also observed that E-cadherin mRNA was down-regulated 2.2-, 3.6- and 5.9-fold in *Brm*-null, *Brg1*-KO and DKO tumors, respectively, compared with WT tumors (p-values <0.001; n=10 for each genotype)(Figure 6Ba). By differential staining of the primary tumors, we observed that Brg1 (data not shown) and E-cadherin loss was greater in the metastatic foci (Figure 6Bb–6Bd). Similarly, Brg1 loss in the *Brg1*-KO tumors was also correlated with the up-regulation of Vimentin, which is another gene that is known to be regulated *in vitro* by SWI/SNF and that is also associated with epithelial-mesenchymal transition (EMT) (Supplementary Figure 3). Therefore, this tumor model recapitulates human lung cancer as it yields distant metastatic foci, which correlates with the down-regulation of E-cadherin and the up-regulation of Vimentin.

**Figure 7:**
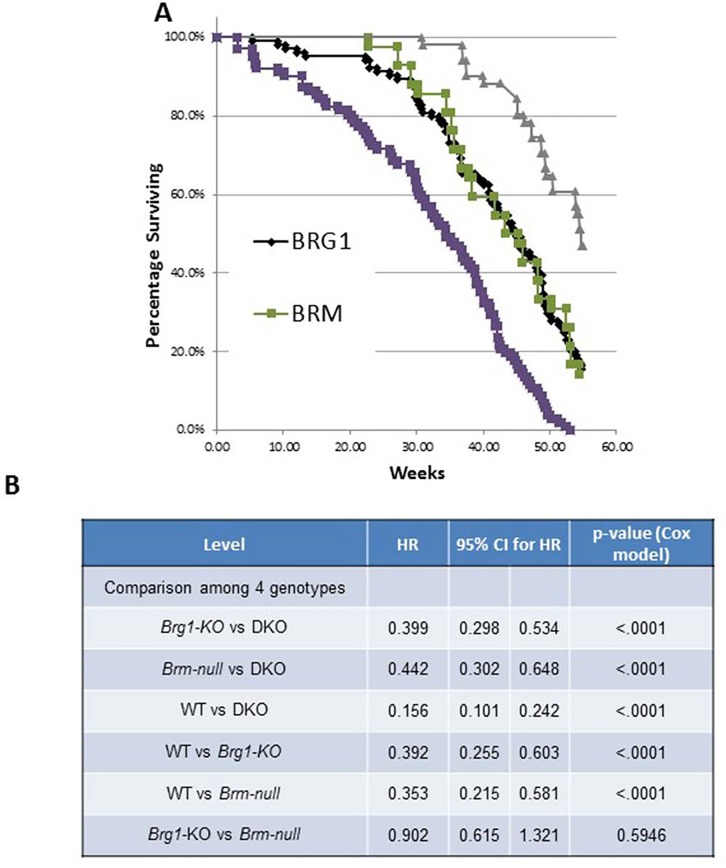
**A.** illustrates the rate of tumor development of lung adenocarcinomas from each of the four genotypes over the 60-week experimental period. **B.** shows the comparisons of various genotypes along with the hazard ratios (HR) and 95% confidence intervals (CI). The p-values obtained from a Cox proportional hazard model are also given.

We also examined mouse survival as a function of genotype and observed that Brg1/Brm loss shortened the latency and increased the frequency of tumor development. To this end, we found that ∼99% of DKO mice succumbed to their tumors prior to the termination of the experiment (Figure 7A). In comparison, according to gross inspection and histological examination, 83% of *Brg1*-KO, 85% of *Brm*-null, and 54% of WT mice developed tumors. Figure 7A illustrates that the time needed for 50% of the DKO, *Brg1*-KO, *Brm*-null and WT genotypes to develop tumors (mean time rate) was 35, 43, 45 and 53 weeks, respectively. The pairwise observed tumor rates were all statistically significant (p<0.0001, Cox hazard ratio) except for the rate in the *Brg1*-KO mice compared with the rate in the *Brm*-null mice (p>0.05) (Figure 7B). The concomitant inactivation of *Brg1* and *Brm* resulted in a shorter latency of tumor development as well as an increase in the percentage of tumor-bearing mice. These data help to illustrate the significant impact that Brg1 and Brm loss has on tumor development, progression and metastasis.

## DISCUSSION

SWI/SNF has been linked to cancer since the discovery that BAF47 is inactivated due to a combination of mutations and deletions in human rhabdoid tumors and the subsequent discovery that *Baf47* was found to induce malignant tumors in a relatively short period of time in mice (∼10 weeks). In contrast, the knockout of either Brg1 or Brm has not yielded robust malignant phenotypes [15, 17, 35]. Considering the homology between the two ATPases (75%), they may exhibit some degree of functional redundancy [13]. In support of this concept, in *in vitro* studies where strong promoters have been used to drive the ectopic expression of these genes, their functions appear to partially overlap. For example, the re-expression of both proteins allows them to bind and cooperate as cofactors for Rb in the restoration of Rb-mediated growth inhibition [36, 37]. Similarly, both proteins induce CD44 and other genes upon transfection into *BRG1/BRM*-deficient cell lines [36, 37], which indicates that in some instances, BRM or BRG1 can substitute for the other. However, other data indicate that, in certain situations, BRG1 and BRM have distinct functions. BRG1 and BRM have been shown to bind to different promoters via their interaction with zinc finger and ankyrin repeat transcription factors, respectively [38]. Therefore, while both proteins seemingly exhibit mostly separate functions, the expression of one protein can partially compensate for the loss of the other. qPCR data from tumors of each of the four genotypes showed in most cases (Tables 1 and 2) that both BRG1 and BRM contribute to the regulation of certain genes. However, the loss of both ATPases results in maximal changes in gene expression. Hence, their overlapping functions could potentially explain why a knockout of either *Brg1* or *Brm* alone does not generate a strong tumorigenic phenotype. According to our data, the dual loss of Brg1 and Brm leads to more aggressive and malignant tumors compared with the loss of Brg1 or Brm alone. Analysis by qPCR of various SWI/SNF-dependent genes shows that some genes are primarily regulated by Brg1, Brm or both equally. In most cases examined, the greatest gene regulation was accomplished by the loss/gain of both Brg1 and Brm, which helps to explain why the most malignant phenotype was seen with the dual loss of BRG1 and BRM together.

Mouse tumor systems can be useful models to study human cancers, especially when they closely mimic the pathogenesis and progression of human tumors. In this respect, the majority of murine lung tumor models commonly yield benign adenomas, while more aggressive adenocarcinomas that show vascular invasion or that yield metastatic lesions seldom arise from most GEMMs. This lack of a metastatic phenotype has been a limitation with most models used to date [39]. Kwon and Berns reviewed murine lung cancer models in 2005 and again in 2013 and found that 0/25 and 2/14, murine model systems, respectively, were noted to yield metastatic phenotypes [40, 41]. In one study, Ji *et al*. combined *Lkb1* inactivation with *Kras* activation and found that mice with the *Kras Lkb1*−/− phenotype developed metastases to intraparenchymal lung lymph nodes (equivalent to stage 2 human lung cancer) at a rate of 50-60%; however, fewer than 4% of mice developed distal metastases outside of the thoracic cavity [42]. Winslow *et al.* showed that missense *Kras* activation/*Tp53* homozygous inactivation led to the development of metastasis of the primary tumors in 2-10% of mice [43]. Similar results were reported in a study by Zheng *et al.* where 36% of the mice with the *Tp53* R172HDg/+ *Kras*LA1/+ phenotype developed metastases; however, metastatic tumors were not found in mice with a similar phenotype of p53 +/−Kras LA1/+ [44]. Interestingly, in the current study, the vast majority of mice that developed distal metastatic disease had tumors that were Brg1-deficient. IHC of the primary and metastatic lesions showed diminutive expression of Brg1, the concomitant loss of E-cadherin expression, and the up-regulation of Vimentin, which together, fosters metastatic behavior as well as EMT. Moreover, the SWI/SNF complex is known to regulate other extracellular proteins as well as cell adhesion proteins, which may also explain how the loss of Brg1 fosters the development of metastatic lesions. It is important to note that we were required to euthanize animals at the first sign of distress. This approach differs from that used in humans who are treated upon their initial presentation (not euthanized), which results in a prolonged survival and continued evolution and spread of their cancers. Moreover, most human metastatic disease is incidentally detected via CT, bone, MRI or PET scans, unlike in our analysis, in which we only detected metastatic spread by gross examination and confirmation by microscropic analysis. Hence, it likely that if the animals were not immediate euthanized and if instead, these mice were analyzed using CT or PET scans, we would have detected a much higher rate of metastatic disease in this model system.

SWI/SNF is known to be a required cofactor for a diverse cadre of key cellular proteins and transcription factors. Many of these proteins are functionally dependent on SWI/SNF, where the impairment or abrogation of Brg1 and/or Brm results in significant changes in gene expression. One aim of this study was to determine which genes would become dysregulated and how changes in their expression contribute to cancer development after Brg1/Brm silencing. We performed a comparative microarray and a qPCR analysis of tumors derived from wild type and DKO mice. The microarray experiment demonstrated that about 6% of the murine genome is regulated at least 2 fold which is consistent with previously reported microarray data from other species. We indexed ∼800 that were regulated >2 fold to determine which categories of genes were inflby Brg1/Brm loss. This showed a broad but distinct change in gene expression where genes that inhibit tumorigenesis (differentiation, tumor suppressors, DNA repair, adhesion) were generally down-regulated while those that promote cancer were up-regulated (proliferation/oncogenes, anti-apoptosis, angiogenesis, metastasis). In particular, loss of SWI/SNF activity impact metabolism and transportation of substance. Moreover, the qPCR analysis of tumors from each of the four genotypes allowed us to explore whether the mRNA expression of specific cancer-related genes was regulated by Brg1, Brm or both. Overall, SWI/SNF affects cancer development and progression in many different ways, especially if and when both *Brg1* and *Brm* are silenced together.

It is critical to realize that only *Brg1* was completely inactivated in this model and that this inactivation occurred via the removal of two exons required for Brg1's helicase domain function. Brm- deficiency was induced by the disruption of one N-terminal exon, which is involved in protein binding; this caused an alternatively spliced *Brm* isoform to be expressed albeit at very low levels. It is clear for *in vitro* work that the double inactivation of *Brg1* and *Brm* is most often lethal in untransformed cells. This likely parallels human cancer where *BRM* is very infrequently mutated, and much more often epigenetically but reversibly silenced in cancer. In fact, no cell lines and likely no primary tumors harbor inactivating mutations in both *BRG1* and *BRM*. As such, this incomplete inactivation of *Brm* is likely the only way we could examine how the loss or depletion of Brg1 affects tumorigenicity. These data have become important in the pursuit of the synthetic lethality concept, in which Brm is experimentally inactivated in Brg1-deficient tumors. Therefore, any drug or other type of approach, which only diminishes but does not completely abrogate Brm activity, will likely lead to a much more tumorigenic phenotype. However, if *Brm* is completely inactivated, it is feasible that tumor cell death might occur.

## MATERIALS AND METHODS

### Initiation of mouse tumors and *Brg1* inactivation

At 6-7 weeks of age, all mice were given 2 intra-peritoneal (IP) injections (1 week apart) of 1 g/kg urethane (ethyl carbamate) to initiate tumor development. These injections also served to prevent apoptosis caused by Cre- mediated *Brg1* inactivation in normal lung cells (i.e., in type 2 alveolar and Clara cells) [18]. Four weeks after the first IP injection, the mice were provided *ad libitum* with water containing 1 mg/mL tetracycline and 3% sucrose for 5 days to induce Cre expression and thus inactivate the *Brg1* allele. Targeted *Brg1* inactivation was localized to lung tissue by the use of a lung-specific promoter (CCSP: Clara cell secretory protein) to drive the tetracycline-sensitive transcription factor (rtTA reverse tetracycline transactivator), which binds to the Cre promoter and drives Cre expression when tetracycline is administered. The expression of Cre recombinase caused the removal of two internal *Brg1* exons flanked by LoxP sites within the catalytic helicase domain, which resulted in the inactivation of *Brg1* (Figure 1A–1C). The *Brm*-null allele was generated by homologous recombination via an insertion of a Neomycin construct into exon 4 of *Brm* [15].

### Gross analysis of the mouse tumors

Over the course of this experiment, we sacrificed the mice only after they exhibited signs of distress as defined by the University of Florida Institutional Animal Care and Use Committee (IACUC). As such, the experimental endpoint was severe physical distress (near death) caused by tumor progression, as demonstrated by weight loss >10%, lack of movement, disheveled appearance, and tachypnea as observed by twice-daily monitoring. The mice were euthanized and subjected to dissection based upon their deviation from normal mouse activity including a decline in body condition. To determine the differentiation status of each of these tumors, a cytology score (as defined by the percentage of tumor cells with high grade tumor cytology, which is characterized by a high nuclear to cytoplasmic ratio, atypical nuclei, prominent nucleoli, and frequent mitotic figures) was calculated by estimating the percentage of cells with the aforementioned characteristics: 0%, 0-30%, 30-70% or >70%; each percentage range was then given a numerical score of 0, 1, 2 or 3, respectively.

### Immunohistochemistry and immunofluorescence

Hematoxylin & eosin (H&E) staining was performed to assess the general histology of the tumors and for scoring purposes. Antibodies to the following antigens were used in IHC/Immunofluorescence (IF) experiments: Brg1 (sc-374197, 1:50, Santa Cruz Biotechnology, Dallas, TX, USA); Brg1 (21634-1-AP, 1:250, Protein Tech, Chicago, IL, USA); Brm (1:200; rabbit polyclonal antibody generated by the Reisman Lab); Pcna (610664, 1:300, BD Biosciences, San Jose, CA, USA); Ki-67 (550609, 1:100, BD Biosciences); p53 (sc-6243, 1:50, Santa Cruz Biotechnology); E-cadherin (GTX61823, 1:100, GeneTex, Irvine, CA, USA); Vimentin (GTX100619, 1:100, GeneTex). All antibodies were tested for cross-reactivity (specificity) by staining cell lines that lack the antigen of interest; these tests were then confirmed by western blotting. All tissue sections were subjected to antigen retrieval, which consisted of heating in a microwave for 15 minutes on the high setting using either 10 mM sodium citrate buffer (pH 6), 10 mM Tris buffer (pH 10) or 10 mM Tris, 1 mM EDTA and 0.05% Tween20 (pH 8), depending on the antibody. Slides were incubated either overnight at 4°C or for 2 hours at room temperature. The appropriate biotinylated secondary antibodies were then used at a 1:200 dilution (BA-1000 or BA-9200, Vector Labs, Burlingame, CA, USA). This was followed by incubation with horseradish peroxidase streptavidin for 1 hour at room temperature (SA-5004, 1:200, Vector Labs). DAB was used as the chromogen (550880, BD Pharmingen, San Jose, CA, USA), and Harris hematoxylin was used as the counterstain.

### Microarray

An 18-microarray chip was used;mRNA samples from 6 DKO and 3 WT tumors each in duplicate were processed at the Sanford Burnham Analytical Genomic core facility and hybridized to a whole transcript (WT) array (Mouse Gene 2.0 ST). Q-PCR was done in triplicate on mRNA derived from 3 match pair of wild type and DKO tumors. The data from the wild type and DKO mice were averaged and subtracted to yield the delta CT values for each gene. We therefore compared the value obtained by the microarray to that obtained by qPCR for 100 genes to determine if the 2-fold and 3-fold levels observed by microarray matched >70% of the qPCR data. Hence, the values obtained by qPCR of 101 genes were compared with the corresponding values obtained by the microarray and were used to validate the number of regulated genes.

### Quantitative real time polymerase chain reaction (qPCR)

For each gene, we conducted qPCR on tumors from 4-5 mice of each of the 4 genotypes (Wild Type (WT), *Brg1/Brm*-knockout (Double-knockout: DKO), *Brg1*- knockout (*Brg1*-KO) and *Brm*-null) in duplicate. We then subtracted the observed values of each gene from those that were obtained from 2 control genes (*ActB* (beta actin) and *Tuba1a* (tubulin 1alpha)). We then averaged the delta CT values that were obtained from each control gene to obtain a delta CT value for each genotype. To determine the relative change in gene expression, we subtracted the delta CT values obtained from the 3 knockout phenotypes: *Brm*-null, *Brg1*-KO or DKO from the delta CT values derived from the wild type tumors. Each of these delta delta CT values was raised to the power of 2 and expressed as the fold change.

### Magnetic resonance imaging (MRI)

Mice were imaged using an Agilent 4.7T MRI instrument located at the Advanced Magnetic Resonance Imaging and Spectroscopy Facility (AMRIS) located at the University of Florida McKnight Brain Institute. Chest cavities were imaged in both the axial and coronal planes. Subsequent images were obtained by ImageJ software (NIH, Bethesda, MD, USA).

## SUPPLEMENTARY FIGURES AND TABLES




