# Supplementary material for: Loss of the SWI/SNF ATPase subunits BRM and BRG1 drives lung cancer development
Source: Oncoscience. 2016 Nov 17;3(11-12):322–36. doi: 10.18632/oncoscience.323 (PMC5235921; doi:10.18632/oncoscience.323)
Supplement: Supplementary file 1 [file oncoscience-03-322-s001.pdf]

Loss of the SWI/SNF ATPase subunits BRM and BRG1 drives lung cancer development

SUPPLEMENTARY FIGURES AND TABLES

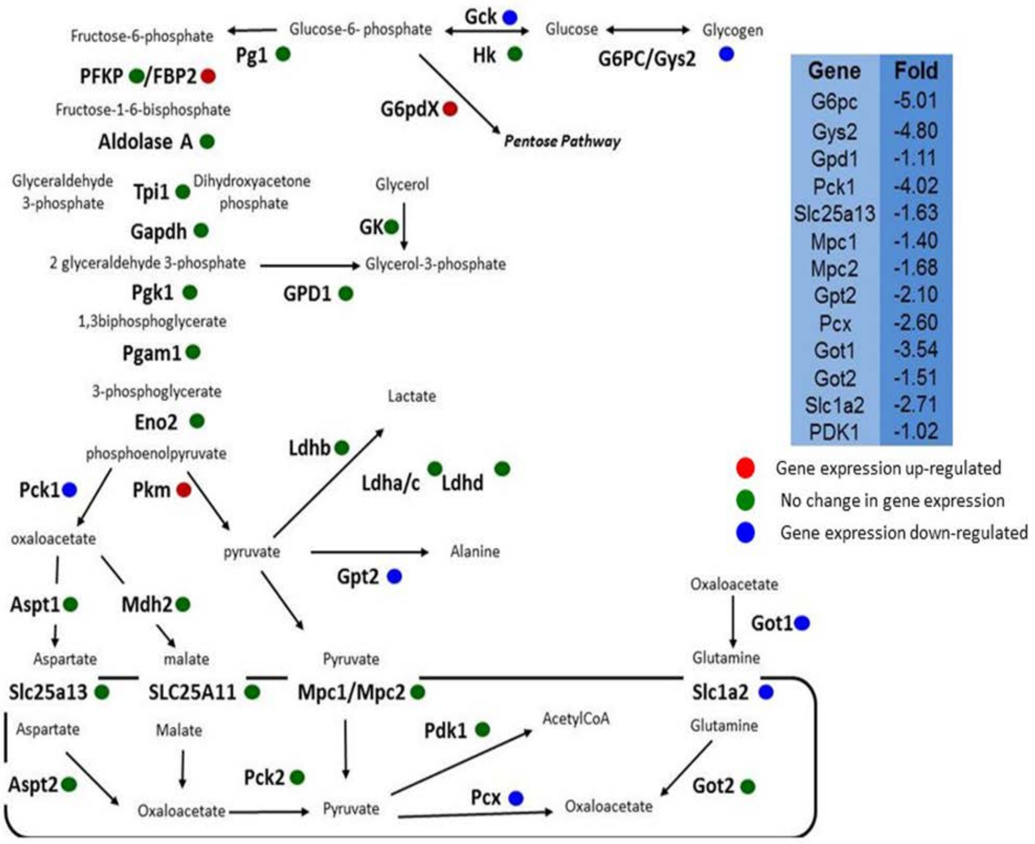

Gene	Fold
G6pc	-5.01
Gys2	-4.80
Gpd1	-1.11
Pck1	-4.02
Slc25a13	-1.63
Mpc1	-1.40
Mpc2	-1.68
Gpt2	-2.10
Pcx	-2.60
Got1	-3.54
Got2	-1.51
Slc1a2	-2.71
PDK1	-1.02

Supplementary Figure S1: Diagram of the enzymes within the main metabolic pathways that are regulated by SWI/SNF greater than 2-fold.

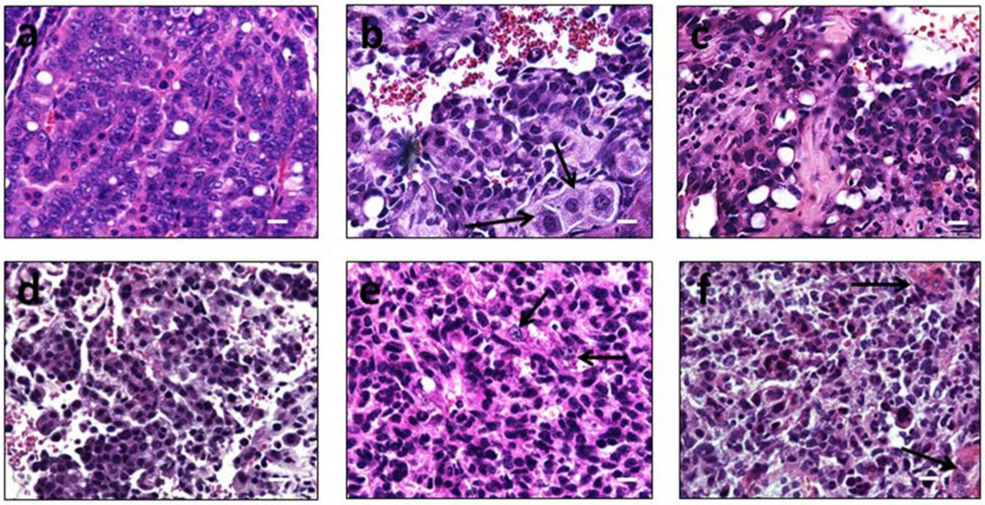

Supplementary Figure S2: Primary lung tumors and distant metastases. a. lung tumor from a DKO mouse a resembles metastases in the liver **b.** and kidney **c.** Cells in a primary lung tumor from a second DKO mouse resembles the cells observed in a liver metastasis **d.** cells in a primary lung tumor from a second DKO mouse resemble the cells observed **e.** and a kidney metastasis **f.** Hepatocytes are also visible among the tumor cells (arrows) in (b) and (e). Normal renal tubule cells (arrows) are visible in (f). Magnification bar = 20 µM.

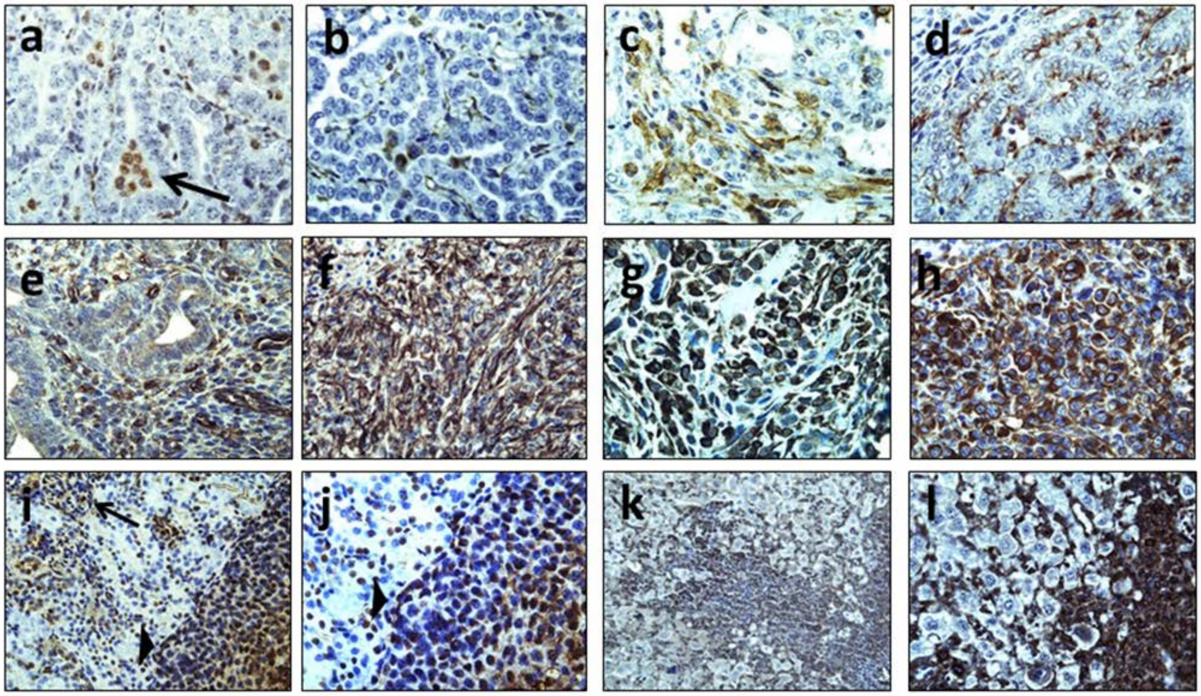

Supplementary Figure S3: Vimentin IHC in primary lung tumors. Lung tumors from a WT and *Brm*-null mouse **a.** and **b.** have qualitatively less vimentin staining than tumors from *Brg1*-KO and DKO mice **c.** and **d.** Note that the macrophages in (a) (arrows) are positive for vimentin. **e.** and **f.** are derived from a *Brg1*-KO (60x) colon. **g.** is derived from a DKO rib met; **h.** is a rib met from a *Brg1*-KO (60x); **i** and **j.** are low and high mag, respectively, of a kidney metastasis from a *Brg1*-KO mouse (20 and 60x); the arrow points to a normal glomerulus, while the arrowhead indicates encroaching tumor cells. **k** and **l.** show a low and high mag, respectively, of a liver metastasis from a DKO mouse.

Supplementary Table S1: An 18-microarray chip was used to determine the differential gene expression from adenocarcinomas derived from wild type and DKO mice. mRNA samples from 6 DKO and 3 WT tumors each in duplicate were processed at the Sanford Burnham Analytical Genomic core facility and hybridized to a whole transcript (WT) array (Mouse Gene 2.0 ST). The data from the wild type and DKO mice were averaged and subtracted to yield the delta CT values for each gene. Data was RMA normalized and log2-transformed. Empirical Bayes moderated t-statistics were used for differential tests. The fold difference and p values are showed. Positive denotes DKO up-regulated compared to WT and negative denotes DKO down-regulated compared to WT.

See Supplementary File 1

Supplementary Table S2: Twenty-five genes that are included in the Foundation One assay to determine the genomic profiles of human cancers were found to be regulated by BRG1 and or BRM according to our microarray dataset

Gene Name	Aliases	Function	Other Functions	Up/Down	Fold Change
CDH1	Arc-1, ECAD	adhesion		down	1.30
ETV5	ERM	adhesion		down	1.99
BCL2	--	apoptosis		down	1.10
BCL2L1	bcl-xL, BCLX	apoptosis		down	1.05
HSD3B1	SDR11E1	development		up	1.84
RUNX1	AML1, CBFA2	development		down	1.11
IGF2	Peg2	development	growth	down	1.52
ETV1	ER81	differentiation	angiogenesis, growth	down	1.02
ROS1	MCF3, c-ros-1	differentiation	growth	down	3.54
GATA4	ASD2, TACHD	differentiation	progression	up	1.37
CDKN1A	WAF1, p21CIP1; CIP1	growth		down	1.36
CDKN1B	CDKN4, P27KIP1	growth		down	1.28
CDKN2B	INK4B, p15INK4b	growth		down	1.12
HGF	TCFB, HPTA	growth		up	2.77
CD79B	AGM6, IGB	immunity		down	1.37
TSHR	CHNG1, LGR3	metabolism		down	1.35
PRSS8	CAP1, PROSTASIN	metastasis	development	down	1.96
MAGI2	ARIP1, SSCAM	metastasis	proliferation	up	1.22
MYCL1	L-MYC	oncogene		up	1.43
NKX2-1	TEBP, TITF1	oncogene		up	1.21
AR	DHTR, HUMARA, HYSP1	signaling		down	2.48
EGFR	ERBB1, HER1	signaling		up	3.16
ESR1	ER, ESTRR	signaling		up	2.08
INPP4B	--	tumor suppressor		down	1.67
SLIT2	SLIL3, Drad-1	tumor suppressor		down	1.80

The human gene name is given along with mouse and or human aliases (when applicable). This illustrates that SWI/SNF regulates the expression of a variety of genes that play a role in tumor development, progression and metastasis and that may impact treatment decisions and a patient's response to therapy.
